# A basic problem of $(p,q)$-Bernstein-type operators

**DOI:** 10.1186/s13660-017-1413-0

**Published:** 2017-06-17

**Authors:** Qing-Bo Cai, Xiao-Wei Xu

**Affiliations:** 1 School of Mathematics and Computer Science, Quanzhou Normal University, Quanzhou, China; 2 School of Mathematical Sciences, Xiamen University, Xiamen, China

**Keywords:** 41A50, $(p,q)$-integer, Bernstein-type approximation, convergence theorem, equivalent condition

## Abstract

In this note, we give an elaboration of a basic problem on convergence theorem of $(p, q)$-analogue of Bernstein-type operators. By some classical analysis techniques, we derive an exact class of $(p_{n},q_{n})$-integer satisfying $\lim _{n\to\infty }[n]_{p_{n},q_{n}}=\infty$ with $\lim _{n\to\infty}p_{n}=1$ and $\lim _{n\to\infty}q_{n}=1$ under $0< q_{n}< p_{n}\leq1$. Our results provide an erratum to corresponding results on $(p,q)$-analogue of Bernstein-type operators that appeared in recent literature.

## Introduction

During the last decades, the applications of *q*-calculus emerged as a new area in the field of approximation theory. The rapid development of *q*-calculus has led to the discovery of various generalizations of Bernstein polynomials involving *q*-integers. A detailed review of the results on *q*-Bernstein polynomials along with an extensive bibliography is given in [1]. The *q*-Bernstein polynomials are shown to be closely related to the *q*-deformed binomial distribution [2]. It plays an important role in the *q*-boson theory giving a *q*-deformation of the quantum harmonic formalism [3]. The *q*-analogue of the boson operator calculus has proved to be a powerful tool in theoretical physics. It provides explicit expressions for the representations of the quantum group $SU_{q}(2)$ [4]. Meanwhile, the $(p,q)$-integers were introduced in order to generalize or unify several forms of *q*-oscillator algebras well known in the earlier physics literature related to the representation theory of single parameter quantum algebras [5].

Recently, $(p,q)$-integers have been introduced into classical linear positive operators to construct new approximation processes. A sequence of $(p,q)$-analogue of Bernstein operators was first introduced by Mursaleen [6, 7]. Besides, $(p,q)$-analogues of Szász-Mirakyan [8], Baskakov Kantorovich [9], Bleimann-Butzer-Hahn [10] and Kantorovich-type Bernstein-Stancu-Schurer [11] operators were also considered, see [12–15]. For further developments, one can also refer to [8, 16–18]. These operators are double parameters corresponding to *p* and *q* versus single parameter *q*-Bernstein-type operators [1, 19, 20]. The aim of these generalizations is to provide appropriate and powerful tools to application areas such as numerical analysis, computer-aided geometric design and solutions of differential equations (see, e.g., [21]).

For example, consider the $(p,q)$-analogue of the Bernstein operators proposed in [7]. Given $f\in C[0,1]$ and $0< q< p\leq1$, operators $B_{n,p,q}$ are defined as follows:

1.1 $$ \begin{aligned}[b] &B_{n,p,q}(f;x) \\ &\quad:=\frac{1}{p^{\frac{n(n-1)}{2}}}\sum_{k=0}^{n}\left [ \textstyle\begin{array}{c} n\\ k \end{array}\displaystyle \right ]_{p,q}p^{\frac{k(k-1)}{2}}x^{k} \prod_{s=0}^{n-k-1} \bigl(p^{s}-q^{s}x \bigr)f \biggl(\frac{[k]_{p,q}}{p^{k-n}[n]_{p,q}} \biggr),\quad n=1,2,\ldots, \end{aligned}$$

where, for any nonnegative integer *k* and $0< q< p\leq1$, the $(p,q)$-integer $[k]_{p,q}$ is defined by

$$[k]_{p,q}:=p^{k-1}+p^{k-2}q+\cdots+q^{k-1}= \frac{p^{k}-q^{k}}{p-q} \quad(k=0,1,2,\ldots), \qquad[0]_{p,q}:=0, $$

and the $(p,q)$-factorial $[k]_{p,q}!$ is defined by

$$[k]_{p,q}!:=[1]_{p,q}[2]_{p,q}\cdots[k]_{p,q}\quad(k=1,2, \ldots),\qquad[0]_{p,q}!:=1. $$

For integers *k*, *n* with $0\leq k\leq n$, the $(p,q)$-binomial coefficient is defined by

$$\left [ \textstyle\begin{array}{c} n\\ k \end{array}\displaystyle \right ]_{p,q}:= \frac{[n]_{p,q}!}{[k]_{p,q}![n-k]_{p,q}!}. $$

In general, we expect $B_{n,p,q}(f;x)$ to converge to $f(x)$ as $n\to \infty$. But we see that, for fixed value of *p* and *q* with $q\in (0,1)$ and $p\in(q,1]$,

$$[n]_{p,q}\to0\quad \text{or}\quad 1/(1-q)\quad \text{as }n\to\infty. $$

To obtain a sequence of generalized $(p,q)$-analogue Bernstein polynomials which converge, we let $q_{n}\in(0,1)$ and $p_{n}\in(q_{n},1]$ depend on *n*. We then choose a sequence $(p_{n},q_{n})$ such that $[n]_{p_{n},q_{n}}\to \infty$ as $n\to\infty$, to ensure that $B_{n,p,q}(f;x)$ converge to $f(x)$.

The convergence theorems for $(p,q)$-analogue Bernstein-type operators were established in some recent papers (see [6], Theorem 3.1 (Remark 3.1), [7], Theorem 1, and further reading [10], Theorem 2.2, [14], Theorem 3.1, [12], Theorem 3, and [11], Remark 2.3, see also [9, 15]). For example, Mursaleen [7] gives the following.

### Theorem 1.1

*Let* $0< q_{n}< p_{n}\leq1$ *such that* $\lim _{n\rightarrow\infty }p_{n}=1$ *and* $\lim _{n\rightarrow\infty}q_{n}=1$. *Then*, *for each* $f\in C[0,1]$, $B_{n,p_{n},q_{n}}(f;x)$ *converge uniformly to* *f* *on* $[0,1]$.

All linear positive operators mentioned in the articles cited above require that $\lim _{n\to\infty} [n]_{p_{n},q_{n}}=\infty$; otherwise, these operators do not define approximation processes. However, the claim that both $\lim _{n\to\infty}p_{n}=1$ and $\lim _{n\to\infty}q_{n}=1$ with $0< q_{n}< p_{n}\leq1$ imply that $\lim _{n\to\infty} [n]_{p_{n},q_{n}}=\infty$, in general, is not true. A counterexample is presented below.

### Example 1.2

Let $p_{n}=1-1/\sqrt{n}$, then $[n]_{p_{n},q_{n}}\to0$ for any sequence $\{ q_{n}\}$ satisfying $0<{q_{n}<p_{n}}$. Indeed,

1.2 $$ \begin{aligned}[b] 0&\leq[n]_{p_{n},q_{n}}=p_{n}^{n-1}+p_{n}^{n-2}q_{n}+ \cdots +p_{n}q_{n}^{n-2}+q_{n}^{n-1} \\ &\leq np_{n}^{n-1}=n(1-1/\sqrt{n})^{n-1}\sim ne^{-\sqrt{n}}\to0,\quad n\to\infty. \end{aligned}$$

Later, the author [8] presented a more accurate assertion: Let $q_{n}$, $p_{n}$ such that $0< q_{n}< p_{n}\leq1$ and $q_{n}\to1$, $p_{n}\to1$, $q^{n}_{n}\to a$, $p^{n}_{n}\to b$ ($a< b$) as $n\to\infty$, then $\lim _{n\to \infty}[n]_{p_{n},q_{n}}\to\infty$. It is natural to ask: What is the class of sequences $(p_{n},q_{n})$ satisfying $\lim _{n\rightarrow \infty}[n]_{p_{n},q_{n}}=\infty$ when $\lim _{n\rightarrow\infty }p_{n}=1$ and $\lim _{n\rightarrow\infty}q_{n}=1$ under $0< q_{n}< p_{n}\leq1$? Undoubtedly, this is an important problem. In this note, we will solve this problem in Section 2.

## Main results

For $0< q_{n}< p_{n}\leq1$, set $q_{n}:=1-\alpha_{n}$, $p_{n}:=1-\beta_{n}$ such that $0\leq\beta_{n}<\alpha_{n}<1$, $\alpha_{n}\rightarrow0$, $\beta _{n}\rightarrow0$ as $n\rightarrow\infty$. In the sequel, we use notation $a_{n} \sim b_{n}\text{ $(a_{n},b_{n}>0)$}\Leftrightarrow\lim _{n\to \infty}\frac{b_{n}}{a_{n}}=1$.

First, let us present the following auxiliary proposition.

### Lemma 2.1

*Let* $n\in\mathbb{N}$, *then as* $n\to\infty$ *we have*

2.1 $$ [n]_{p_{n},q_{n}}\to\infty\quad\Rightarrow\quad e^{n\beta_{n}}/n\to0. $$

*On the other hand*,

2.2 $$ e^{n\alpha_{n}}/n\to0\quad\Rightarrow\quad[n]_{p_{n},q_{n}}\to\infty. $$

### Proof

We note that

2.3 $$ \begin{aligned}[b] [n]_{p_{n},q_{n}}&=q_{n}^{n-1}+p_{n}q_{n}^{n-2}+\cdots+p_{n}^{n-2}q_{n}+p_{n}^{n-1} =q_{n}^{n-1}\bigl(1+p_{n}/q_{n}+\cdots+(p_{n}/q_{n})^{n-1} \bigr) \\ &>nq_{n}^{n-1}=n(1-\alpha_{n})^{(-1/{\alpha_{n}})(n-1)(-\alpha_{n})} \sim n/e^{n\alpha_{n}},\quad n\to\infty. \end{aligned}$$

Similarly, we have

2.4 $$ \begin{aligned}[b] [n]_{p_{n},q_{n}}&=q_{n}^{n-1}+p_{n}q_{n}^{n-2}+\cdots+p_{n}^{n-2}q_{n}+p_{n}^{n-1} =p_{n}^{n-1}\bigl(1+q_{n}/p_{n}+\cdots+(q_{n}/p_{n})^{n-1} \bigr) \\ &< np_{n}^{n-1}=n(1-\beta_{n})^{(-1/{\beta_{n}})(n-1)(-\beta_{n})} \sim n/e^{n\beta_{n}},\quad n\to\infty. \end{aligned}$$

Therefore, from (2.3) and (2.4), for sufficiently large *n*, there exist two positive real numbers $C_{1}$ and $C_{2}$ satisfying

2.5 $$ C_{1}\cdot n/e^{n\alpha_{n}}< [n]_{p_{n},q_{n}}< C_{2}\cdot n/e^{n\beta_{n}}. $$

This yields the proof. □

The main result of this work is expressed by the next assertion.

### Theorem 2.1

*The following statements are true*:

($\mathcal{A}$)
*If* $\lim _{n\to\infty}e^{n(\beta _{n}-\alpha_{n})}=1$ *and* $e^{n\beta_{n}}/n\to0$, *then* $[n]_{p_{n},q_{n}}\to \infty$.
($\mathcal{B}$)
*If* $\varlimsup _{n\to\infty }e^{n(\beta_{n}-\alpha_{n})}<1$ *and* $e^{n\beta_{n}}(\alpha_{n}-\beta_{n})\to 0$, *then* $[n]_{p_{n},q_{n}}\to\infty$.
($\mathcal{C}$)
*If* $\varliminf _{n\to\infty }e^{n(\beta_{n}-\alpha_{n})}<1$, $\varlimsup _{n\to\infty }e^{n(\beta_{n}-\alpha_{n})}=1$ *and* $\max\{e^{n\beta_{n}}/n,e^{n\beta _{n}}(\alpha_{n}-\beta_{n})\}\to0$, *then* $[n]_{p_{n},q_{n}}\to\infty$.

*Conversely*,

($\mathcal{B'}$)
*If* $\varlimsup _{n\to\infty }e^{n(\beta_{n}-\alpha_{n})}<1$ *and* $[n]_{p_{n},q_{n}}\to\infty$, *then* $e^{n\beta_{n}}(\alpha_{n}-\beta_{n})\to0$.
($\mathcal{C'}$)
*If* $\varliminf _{n\to\infty }e^{n(\beta_{n}-\alpha_{n})}<1,\varlimsup _{n\to\infty }e^{n(\beta_{n}-\alpha_{n})}=1$ *and* $[n]_{p_{n},q_{n}}\to\infty$, *then* $\max\{e^{n\beta_{n}}/n, e^{n\beta_{n}}(\alpha_{n}-\beta_{n})\}\to0$.

### Proof

Case ($\mathcal{A}$):

If $\lim _{n\to\infty}e^{n(\beta_{n}-\alpha_{n})}=1$, since $\lim _{n\to\infty}e^{n\alpha_{n}}/n=\lim _{n\to \infty}e^{n\beta_{n}}/n/e^{n(\beta_{n}-\alpha_{n})}\to0$ and combined with (2.2) imply $[n]_{p_{n},q_{n}}\to\infty$ (see Remark 2.1).

Case ($\mathcal{B}$) and Case ($\mathcal{B'}$):

If $\varlimsup _{n\to\infty}e^{n(\beta_{n}-\alpha_{n})}<1$. Note that, for sufficiently large *n*,

2.6 $$ [n]_{p_{n},q_{n}}=p_{n}^{n-1}\frac{1-(q_{n}/p_{n})^{n}}{1-q_{n}/p_{n}}\sim \frac {1}{e^{n\beta_{n}}}\frac{1- (1-(\alpha_{n}-\beta_{n})/(1-\beta _{n}) )^{n}}{(\alpha_{n}-\beta_{n})/(1-\beta_{n})}. $$

Since

2.7 $$ \frac{\alpha_{n}-\beta_{n}}{1-\beta_{n}}\to0, $$

it is not difficult to obtain from (2.7) and $\varlimsup _{n\to\infty}e^{n(\beta_{n}-\alpha_{n})}<1$ that, for sufficiently large *n*, there exists $c\in(0,1)$ such that

2.8 $$ \biggl(1-\frac{\alpha_{n}-\beta_{n}}{1-\beta_{n}} \biggr)^{n}\sim e^{n(\beta_{n}-\alpha_{n})}< c. $$

Set $d_{n}:=1- (1-\frac{\alpha_{n}-\beta_{n}}{1-\beta_{n}} )^{n}$, then for sufficiently large *n*, $d_{n}>1-c$. Thus, from (2.6), (2.7) and (2.8), we have

2.9 $$ [n]_{p_{n},q_{n}}\sim\frac{1}{e^{n\beta_{n}}}\cdot\frac{d_{n}}{\alpha _{n}-\beta_{n}}, $$

which entails that

2.10 $$ [n]_{p_{n},q_{n}}\to\infty\quad\Leftrightarrow\quad e^{n\beta_{n}}( \alpha_{n}-\beta _{n})\to0. $$

This yields the proof of Case ($\mathcal{B}$) and Case ($\mathcal{B'}$).

Case ($\mathcal{C}$) and Case ($\mathcal{C'}$):

If $\varliminf _{n\to\infty}e^{n(\beta_{n}-\alpha _{n})}<1$, $\varlimsup _{n\to\infty}e^{n(\beta_{n}-\alpha_{n})}=1$. Since the sequence $0< x_{n}:=e^{n(\beta_{n}-\alpha_{n})}<1$ is bounded, set $E:=\{x|x\text{ is a limit point of }\{x_{n}\},n\geq1\}$, then $\sup E=1$ from $\varlimsup _{n\to\infty}e^{n(\beta_{n}-\alpha _{n})}=1$. Now, we are going to extract a subsequence $\{x_{n_{k}}\}$ of $\{ x_{n}\}$ such that

- $\lim _{k\to\infty}x_{n_{k}}=1$, and
- $E^{1}:=\{x|x\text{ is a limit point of }\{x_{n}\}\setminus\{x_{n_{k}}\}\}$, with $\sup E^{1}<1$.

We verify that it is possible to extract such a subsequence $\{x_{n_{k}}\} $. Since 1 is a limit point of $A:=\{x_{n},n\geq1\}$, take a subsequence $\{x_{n^{(1)}_{k}}\}$ of *A* such that $\lim _{k\to \infty}x_{n^{(1)}_{k}}=1$, set $A^{(1)}:=\{x_{n^{(1)}_{k}},k\geq1\}$ and let $A_{1}:=A\setminus A^{(1)}$. If 1 is also a limit point of $A_{1}$, take a subsequence $\{ x_{n^{(2)}_{k}}\}$ of $A_{1}$ such that $\lim _{k\to\infty}x_{n^{(2)}_{k}}=1$, set $A^{(2)}:=\{x_{n^{(2)}_{k}},k\geq1\}$ and let $A_{2}:=A_{1}\setminus A^{(2)}$. Continuing this process, we obtain a series of sequences, i.e., $A^{(1)},A^{(2)},\ldots$ , set $A_{f}:=A\setminus\bigcup _{s\in\mathbb{I}\subseteq\mathbb{N}}A^{(s)}$. Since *A* is a countable set, this process will stop until 1 is not a limit point of $A_{f}$ after finite or countable steps; otherwise, we will see that 1 is the only limit point of *A*, which contradicts to the assumptions of Case ($\mathcal{C}$). Then we can take the subsequence $\{x_{n_{k}}\}=\bigcup _{s\in\mathbb {N}}A^{(s)}$ which satisfies (a) and (b).

Set $\{x_{n'_{k}}\}:=\{x_{n}\}\setminus\{x_{n_{k}}\}$, it is obvious that $\{ n_{k},k\geq0\}\cup\{n'_{k},k\geq0\}=\mathbb{N}$. Then from ($\mathcal{A}$) and (a), we have seen that $[n]_{p_{n_{k}},q_{n_{k}}}\to\infty\Leftrightarrow e^{n_{k}\beta _{n_{k}}}/n_{k}\to0$ as $k\to\infty$. And since $\varlimsup _{n\to\infty}x_{n'_{k}}<1$ from (b), we also have seen from ($\mathcal{B}$) that $[n]_{p_{n'_{k}},q_{n'_{k}}}\to\infty\Leftrightarrow e^{n'_{k}\beta _{n'_{k}}}(\alpha_{n'_{k}}-\beta_{n'_{k}})\to0$ as $k\to\infty$. In summary,

(i) $\lim _{n\to\infty}[n]_{p_{n},q_{n}}=\infty\Leftrightarrow$ for any subsequence $\{N_{k}\}$ of a natural number set such that $\lim _{k\to\infty}N_{k}=\infty$, we have $\lim _{k\to \infty}[n]_{p_{N_{k}},q_{N_{k}}}=\infty$.
(ii) Since $\{n_{k}\}_{k\geq0}$ and $\{n'_{k}\}_{k\geq0}$ are two subsequences of a natural number set such that $\lim _{k\to\infty}n_{k}=\infty$ and $\lim _{k\to \infty}n'_{k}=\infty$, thus as $k\to\infty$
  $$\lim _{n\to\infty}[n]_{p_{n},q_{n}}=\infty\quad\Rightarrow\quad \textstyle\begin{cases} [n]_{p_{n_{k}},q_{n_{k}}}\to\infty\quad\Leftrightarrow\quad e^{n_{k}\beta_{n_{k}}}/n_{k}\to0, \\ [n]_{p_{n'_{k}},q_{n'_{k}}}\to\infty\quad\Leftrightarrow\quad e^{n'_{k}\beta _{n'_{k}}}(\alpha_{n'_{k}}-\beta_{n'_{k}})\to0. \end{cases} $$

We assert that the inverse proposition of (ii) also holds. Indeed, for each sufficiently large $N>0$, there exists a positive integer $K_{1}$ such that for every natural number $k>K_{1}$, we have $[n]_{p_{n_{k}},q_{n_{k}}}>N$; meanwhile, for the previous $N>0$, there exists a positive integer $K_{2}$ such that for every natural number $k>K_{2}$, we have $[n]_{p_{n'_{k}},q_{n'_{k}}}>N$; take $n_{0}=\max\{ n_{K_{1}},n'_{K_{2}}\}$, for every natural number $n>n_{0}$, we have $[n]_{p_{n},q_{n}}>N$, i.e., $\lim _{n\to\infty }[n]_{p_{n},q_{n}}=\infty$. Now we have proved that as $k\to\infty$

2.11 $$ \lim _{n\to\infty}[n]_{p_{n},q_{n}}=\infty\quad\Leftrightarrow\quad\Delta:= \textstyle\begin{cases} e^{n_{k}\beta_{n_{k}}}/n_{k}\to0, &\text{(c)} \\ e^{n'_{k}\beta_{n'_{k}}}(\alpha_{n'_{k}}-\beta_{n'_{k}})\to0. &\text{(d)} \end{cases} $$

Next, we infer that as $k\to\infty$

2.12 $$ \Delta\quad\Leftrightarrow\quad\lim _{n\to\infty}\max\bigl\{ e^{n\beta _{n}}/n,e^{n\beta_{n}}( \alpha_{n}-\beta_{n})\bigr\} = 0. $$

‘⇐’ of (2.12) is straightforward. Now we show ‘⇒’. On the one hand, by Remark (2.2), we know from (d) of Δ that $e^{n'_{k}\beta_{n'_{k}}}/n'_{k} \to0$, and combined with (c) $e^{n_{k}\beta_{n_{k}}}/n_{k}\to0$, we can show $e^{n\beta_{n}}/n\to0$ by using a similar method as in the previous paragraph. On the other hand, $e^{n_{k}\beta_{n_{k}}}(\alpha_{n_{k}}-\beta_{n_{k}})\to0$ is straightforward (since $\lim _{k\to\infty}x_{n_{k}}=\lim _{k\to\infty}e^{n_{k}(\beta_{n_{k}}-\alpha_{n_{k}})}=1$, and note (c) in Δ), and combined with $e^{n'_{k}\beta_{n'_{k}}}(\alpha _{n'_{k}}-\beta_{n'_{k}})\to0$, we can also deduce that $e^{n\beta _{n}}(\alpha_{n}-\beta_{n})\to0$. This yields the proof of ‘⇒’ in (2.12).

Therefore, ($\mathcal{C}$) and ($\mathcal{C'}$) follow from (2.11) and (2.12). □

### Remark 2.1

In general, from (2.1) we have only $[n]_{p_{n},q_{n}}\to\infty \Rightarrow e^{n\beta_{n}}/n\to0$. However, if $\lim _{n\to \infty}e^{n(\beta_{n}-\alpha_{n})}=1$ holds, then we have the equivalent relation $e^{n\beta_{n}}/n\to0\Leftrightarrow[n]_{p_{n},q_{n}}\to\infty$ from ($\mathcal{A}$). Thus, we have:

($\mathcal{A}_{0}$)
If $\lim _{n\to\infty}e^{n(\beta _{n}-\alpha_{n})}=1$, then $e^{n\beta_{n}}/n\to0\Leftrightarrow [n]_{p_{n},q_{n}}\to\infty$.

Similarly, from ($\mathcal{B}$) and ($\mathcal {B'}$), ($\mathcal{C}$) and ($\mathcal{C'}$) we have

($\mathcal{B}_{0}$)
If $\varlimsup _{n\to\infty }e^{n(\beta_{n}-\alpha_{n})}<1$, then $e^{n\beta_{n}}(\alpha_{n}-\beta _{n})\to0\Leftrightarrow[n]_{p_{n},q_{n}}\to\infty$.
($\mathcal{C}_{0}$)
If $\varliminf _{n\to\infty }e^{n(\beta_{n}-\alpha_{n})}<1$, $\varlimsup _{n\to\infty }e^{n(\beta_{n}-\alpha_{n})}=1$, then $\max\{e^{n\beta_{n}}/n,e^{n\beta _{n}}(\alpha_{n}-\beta_{n})\}\to0\Leftrightarrow[n]_{p_{n},q_{n}}\to\infty$.

### Remark 2.2

In Case ($\mathcal{B}$), we can also deduce directly from $\varlimsup _{n\to\infty}e^{n(\beta_{n}-\alpha_{n})}<1$ and $e^{n\beta_{n}}(\alpha_{n}-\beta_{n})\to0$ that $e^{n\beta_{n}}/n\to 0$ as $n\to\infty$. Indeed, since $\varlimsup _{n\to\infty }e^{n(\beta_{n}-\alpha_{n})}<1$, and combined with the classical inequality on upper (lower) limit

$$\begin{aligned} \varliminf _{n\to\infty}e^{n(\alpha_{n}-\beta_{n})} \cdot \varlimsup _{n\to\infty}e^{n(\beta_{n}-\alpha_{n})}\geq \varliminf _{n\to\infty} \bigl(e^{n(\alpha_{n}-\beta_{n})} \cdot e^{n(\beta_{n}-\alpha_{n})} \bigr)=1, \end{aligned}$$

we have $\varliminf _{n\to\infty}e^{n(\alpha_{n}-\beta _{n})}>1$. Thus, for sufficiently large *n*, there exists $c_{0}>1$ such that $e^{n(\alpha_{n}-\beta_{n})}>c_{0}$. This means that $0<(\log c_{0}) e^{n\beta_{n}}/n<e^{n\beta_{n}}/n\cdot(n(\alpha_{n}-\beta_{n}))\to0$, and we have seen that $e^{n\beta_{n}}/n\to0$.

### Remark 2.3

Now we utilize Theorem 2.1 (Remark 2.1) to elaborate Example 1.2 again. In the example, $\beta_{n}=1/\sqrt {n}$, while $e^{n\beta_{n}}/n=e^{\sqrt{n}}/n \nrightarrow0$ as $n\to \infty$, thus $[n]_{p_{n},q_{n}}\nrightarrow\infty$.

For $p_{n}=1$, $\beta_{n}=0$, $q_{n}=1-\alpha_{n}$, then $e^{n\beta_{n}}/n=1/n\to0$ and $e^{n\beta_{n}}(\alpha_{n}-\beta_{n})=\alpha_{n}\to0$. Thus any case of ($\mathcal{A}_{0}$)-($\mathcal{C}_{0}$) is straightforward. In this case, $(p_{n}, q_{n})$-integer reduces to $q_{n}$-integer, and it is known that $[n]_{q_{n}}\to\infty \Leftrightarrow q_{n}\to1$ as $n\to\infty$. See [19], Theorem 2, and [22], formula (2.7).

## Conclusion

In this note, we mainly obtain the sufficient and necessary conditions for $(p,q)$-integer $[n]_{p,q}$ tending to infinity as $n\to\infty$. The conclusion guarantees the $(p,q)$-analogue of Bernstein-type operators to be approximation processes as $n \to\infty$.
